# A Complex Interaction Between Reduced Reelin Expression and Prenatal Organophosphate Exposure Alters Neuronal Cell Morphology

**DOI:** 10.1177/1759091416656253

**Published:** 2016-06-30

**Authors:** Brian R. Mullen, Brennan Ross, Joan Wang Chou, Rana Khankan, Elvira Khialeeva, Kimberly Bui, Ellen M. Carpenter

**Affiliations:** 1 Molecular, Cellular and Integrative Physiology, University of California, Los Angeles, CA, USA; 2 Neuroscience Interdepartmental Program, University of California, Los Angeles, CA, USA; 3 Integrative Biology and Physiology, University of California, Los Angeles, CA, USA; 4 Molecular Biology Interdepartmental Program, University of California, Los Angeles, CA, USA; 5 Molecular, Cellular and Developmental Biology, University of California, Los Angeles, CA, USA; 6 Department of Psychiatry and Biobehavioral Science, University of California, Los Angeles, CA, USA

**Keywords:** cortex, cerebellum, dendrite, hippocampus, mouse model, pesticide

## Abstract

Genetic and environmental factors are both likely to contribute to neurodevelopmental disorders including schizophrenia, autism spectrum disorders, and major depressive disorders. Prior studies from our laboratory and others have demonstrated that the combinatorial effect of two factors—reduced expression of reelin protein and prenatal exposure to the organophosphate pesticide chlorpyrifos oxon—gives rise to acute biochemical effects and to morphological and behavioral phenotypes in adolescent and young adult mice. In the current study, we examine the consequences of these factors on reelin protein expression and neuronal cell morphology in adult mice. While the cell populations that express reelin in the adult brain appear unchanged in location and distribution, the levels of full length and cleaved reelin protein show persistent reductions following prenatal exposure to chlorpyrifos oxon. Cell positioning and organization in the hippocampus and cerebellum are largely normal in animals with either reduced reelin expression or prenatal exposure to chlorpyrifos oxon, but cellular complexity and dendritic spine organization is altered, with a skewed distribution of immature dendritic spines in adult animals. Paradoxically, combinatorial exposure to both factors appears to generate a rescue of the dendritic spine phenotypes, similar to the mitigation of behavioral and morphological changes observed in our prior study. Together, our observations support an interaction between reelin expression and chlorpyrifos oxon exposure that is not simply additive, suggesting a complex interplay between genetic and environmental factors in regulating brain morphology.

## Introduction

The contribution of both genetic and environmental factors to neurodevelopmental disorders such as autism spectrum disorders (ASD) and schizophrenia (SZ) has been recognized for many years. Heritability of these disorders provides evidence for a genetic component, and a variety of genetic factors have been identified that influence the onset and progression of these diseases (Petronis, 2004; Persico and Bourgeron, 2006; Abrahams and Geschwind, 2008; Loke et al., 2015). However, a number of environmental risk factors, including dietary factors, maternal diabetes, prenatal and perinatal stress, parental age, prenatal exposure to drugs and medications, zinc deficiencies, nutritional supplements, pesticide exposure, urban air pollution, heavy metal toxins, and maternal infection have also been implicated in these disorders (Murray et al., 1992; DeSoto and Hitlan, 2010; Freire et al., 2010; Herbert, 2010; Shelton et al., 2012; Christensen et al., 2013; Cohen et al., 2013; Koufaris and Sismani, 2015), leading to the idea that both genetic and environmental factors contribute to neurodevelopmental disorders.

Although a combination of genetic and environmental factors is likely the basis for many neuropsychiatric disorders, how these gene X environment interactions affect cellular structure or function is poorly understood. In the current study, we examined the effects of two potential contributing factors to neuropsychiatric disorders—reduced expression of the secreted signaling protein reelin and prenatal exposure to an organophosphate pesticide, chlorpyrifos oxon (CPO)—on cellular morphology in the adult mouse brain. Reelin signaling is widely recognized as an important factor in normal brain development, and loss of this signaling leads to lamination defects and neuronal disorganization in the cortex, cerebellum, hippocampus, and other areas, along with altered dendritic spine formation and reduced dendritic branching (Hong et al., 2000; Rice and Curran, 2001; Chang et al., 2007; Zaki et al., 2007; Niu et al., 2008; O’Dell et al., 2015). Although complete loss of reelin signaling is rare in humans, reduced reelin expression has been implicated in multiple neuropsychiatric disorders including ASD, SZ, epilepsy, and depression (Guidotti et al., 2000; Fatemi et al., 2005; Chow et al., 2012; Dazzo et al., 2015). Exposure to organochlorine and organophosphate agricultural pesticides has been correlated with an increased prevalence of ASDs (Roberts et al., 2007) and mutations that disrupt PON1, which encodes an organophosphate inactivator, show significant association with autism (D’Amelio et al., 2005).

Previous studies from our laboratory and others have demonstrated paradoxical effects of combining reduced reelin and CPO exposure on ASD-related behaviors (Laviola et al., 2006; Mullen et al., 2013), such that either reduced reelin or prenatal CPO exposure produced alterations in communication and social behavior, but both factors together produced an amelioration of the behaviors. Our prior study also revealed cell positioning and layering defects that resulted from either reduced reelin or CPO exposure, but combinatorial exposure to both factors restored normal anatomical organization (Mullen et al., 2013). In the current study, we examined cell distribution, cellular morphology, and dendritic spine organization in the cortex, hippocampus, and cerebellum to determine if the combination of reduced reelin and CPO exposure directly affected cellular morphology. Using GAD67-GFP mice to identify GABAergic cells, which are the cell type that expresses reelin in adult animals, we found little difference in cell distribution in the cortex. We found that reduced reelin expression and CPO exposure altered spine morphology in both the hippocampus and cerebellum. We demonstrate that reduced reelin alone is sufficient to decrease overall spine density and to affect one specific class of spines, the mushroom spines. We also show that CPO exposure alone does not affect spine density but does increase the ratio of immature to mature spines. The combination of reduced reelin and CPO exposure appears to generate a phenotype highly similar to CPO exposure alone, suggesting that prenatal CPO exposure is sufficient to alter dendritic spine morphology throughout life and that this effect may be independent of any residual effects on reelin protein levels.

## Materials and Methods

### Mouse Lines and Minipump Implantation

All animal studies were conducted in accordance with the UCLA Office of Animal Research Oversight and Institutional Animal Care and Use Committee protocols. To determine the effects of reduced reelin expression and CPO exposure on gross morphology, protein expression, and dendritic spine distribution, C57Bl/6 female mice were crossed with heterozygous male *reeler* mice (B6C3Fe-a/a-*Reln^Rl/+^*) originally obtained from the Jackson Laboratory (Bar Harbor, ME, USA); these mice are referred to as *Rl^+/^^−^* throughout the text. At gestational Day 13.5, pregnant females were implanted with osmotic minipumps loaded with 6 mg/ml (20 mg/kg) of CPO (or a vehicle control) as previously described (Mullen et al., 2013). This dose corresponds to that used by other groups (Laviola et al., 2006) and is well below the reported LD50 of 60 mg/kg (NPIC Fact Sheet, http://npic.orst.edu/factsheets/archive/chlorptech.html); 20 mg/kg corresponds to a moderate environmental exposure for humans. In total, 10 pregnant mice were used for minipump implantation. Females delivered their litters at approximately gestational Day 20. Pups were raised by their dams to P28, then weaned and group housed until used for anatomical and biochemical studies at postnatal day (P) 90. Four groups of animals were used for analysis—vehicle-treated *Rl^+/+^* and *Rl^+/^^−^* mice and CPO-treated *Rl^+/+^* and *Rl^+/^^−^* mice. Only male mice were used for these studies to eliminate complications resulting from female hormonal status, and no more than one animal per litter was included in each group.

To determine the effects of CPO on interneuron populations, male GAD67*^GFP+/^^−^* C57Bl/6 mice (Tamamaki et al., 2003) were crossed with heterozygous female *reeler Orleans (Rl^rl^^−^^Orl/+^)* Balb/C mice (gift of Dr. PE Phelps, UCLA; Abadesco et al., 2014). *Rl^rl^^−^^Orl/+^* produce 50% normal reelin and 50% mutated protein that is manufactured but not secreted (de Bergeyck et al., 1997). Pregnant mice were implanted with osmotic minipumps as described earlier at G13.5. GABAergic interneurons migrate into the cerebral cortex and hippocampus at embryonic day (E) 11.5–E16.5 (Anderson et al., 2001), thus minipump implantation corresponded to a stage of active interneuron migration. Offspring from the minipump-implanted dams were visually assessed for the expression of the GFP protein using UV illumination at P1-3. Genotypes of the *reeler Orleans* allele were determined using PCR as described (Hammond et al., 2006).

### Western Blotting for Reelin Fragment Quantification

Adult brains were rapidly dissected out of P90 animals (*Rl^+/+^* Veh, *n* = 4; *Rl^+/+^* CPO, *n* = 4; *Rl^+/^^−^* Veh, *n* = 4; *Rl^+/^^−^* CPO, *n* = 4). One hemisphere was collected and separated to isolate the neocortex, hippocampus, and the remaining components of the brain; the remaining hemispheres and cerebella were used for Golgi analysis (see below). Tissue samples were ﬂash frozen in a dry ice-ethanol bath and stored at −80℃. Protein bands were resolved as described in Mullen et al. (2013).

### Histology and Immunohistochemistry

#### Golgi staining

Single brain hemispheres and cerebella were stained utilizing an FD Rapid GolgiStain Kit (FD NeuroTechnologies). The cerebral hemispheres were separated from the cerebellum for staining. The brains were placed into Golgi-Cox solution for 2 weeks at room temperature, protected from the light. The cerebella were sagittally sectioned and the cerebral hemispheres were coronally sectioned at 200 µm on an oscillating tissue slicer (Electron Microscopy Sciences). Sections were collected in phosphate-buffered saline (PBS) then mounted on gelatin-coated glass microscope slides. After mounting, the sections were reacted with the silver–mercury solutions provided in the kit and cover-slipped using Permount (Fisher).

#### Nissl staining

Coronal and sagittal 30 µm frozen sections were collected from P90 mice (*Rl^+/+^* Veh, *n* = 3; *Rl^+/+^* CPO, *n* = 4; *Rl^+/^^−^* Veh, *n* = 3; *Rl^+/^^−^* CPO, *n* = 4) and stained with cresyl violet. The slides were viewed and photographed using a Zeiss Axioskop with a cooled CCD camera; quantification and analysis were performed using ImageJ software.

#### DAPI staining

Coronal frozen sections (30 µm) were collected and air-dried onto Superfrost Plus slides. Slides were rinsed briefly with PBS and then stained for 5 min at room temperature with 300 nM DAPI (Life Technologies). Slides were rinsed with PBS, coverslipped with 90% glycerol or PBS, and stored at 4℃.

#### Immunofluorescence

Free floating tissue sections of 30 µm coronal neocortex and hippocampus of *Rl^rl^^−^^Orl/+^*/GAD67 *^GFP+/^^−^* Balb/C treated prenatally with either vehicle or CPO were stained with rat anti-Ctip2 (Abcam; 1:500 dilution), mouse anti-reelin (EMD Millipore; 1:500 dilution), or chicken anti-GFP (Novus; 1:1000 dilution) and counterstained with DAPI. The M1 region of the frontal motor cortex and rostral hippocampus in vehicle-treated tissue (*n* = 3) and CPO-treated tissue (*n* = 4) were examined to determine interneuron subtype. The M1 cortex was subdivided into three regions based on Ctip2 and DAPI staining: Layer I, Layers II to IV, and Layers V to VI. Hippocampi were divided into four regions: dentate gyrus, striatum radiatum, striatum moleculare, and striatum pyramidal or striatum oriens or striatum alveus.

#### Image collection and analysis

DAPI and Nissl-stained sections were images on a Zeiss Axioskop equipped with a cooled CCD camera and Axiovision software. Image analysis and densitometry were performed using NIH ImageJ. Densitometry was performed by creating a column average plot for each section using profile plot on a weighted grayscale value (RGB 0.30, 0.59, 0.11) assigned to each pixel as described in Mullen et al. (2013). Golgi images were collected on a Zeiss Apotome, assembled as Z-stacks and analyzed using Axiovision software and ImageJ. GFP or anti-reelin images were collected on Zeiss LSM 510 Confocal microscope and assessed in Neurolucida.

### Sholl Analysis and Dendritic Spine Characterization

Sholl analysis was performed on camera lucida drawings made of Golgi-stained CA1 pyramidal cells (*Rl^+/+^* Veh, *n* = 21 cells from three animals; *Rl^+/+^* CPO, *n* = 21 cells from four animals; *Rl^+/^^−^* Veh, *n* = 23 cells from three animals; *Rl^+/^^−^* CPO, *n* = 21 cells from four animals). The first Sholl ring was placed 50 µm from the center of the cell body; subsequent rings were placed every 25 µm to a total distance of 300 µm; each ring was scored for the total number of processes crossed.

Spine characterization and classification was performed on z-stack images collected from Golgi-stained material on a Zeiss Apotome microscope in bright field using a 100 × oil immersion objective. Image analysis was performed using AxioVision software (Zeiss). Classifications of dendritic spines were performed in the hippocampus and the cerebellum. In the hippocampus, two dendrites on the CA1 pyramidal cells—the first apical oblique dendrite (AO) closest to the soma and a basal dendrite (Bas) after the first branch point off the soma—were selected for analysis. In the cerebellum, spines were characterized on the distal dendrites of Purkinje cells that were stained in Lobules III, IV/V, VI, and VII. All protrusions off the dendrites between 30 µm and 115 µm in length were classified as mushroom, thin, stubby, branched, or filopodial spines following parameters established by Lee et al. (2004a). Mushroom spines had discernible stalks and heads, thin spines were longer than mushroom spines without a discernible head, stubby spines were as thick as the head of a mushroom spine, branched spines had a bifurcated stalk, and filopodial spines were defined as those thin spines whose width was less than half the width of the dendrite it was protruding from, but longer than the total width of the dendrite. Both total spine length and cross-sectional areas of mushroom heads were measured. The total number of spines examined and classified is presented in Table 1 and the numbers of mushroom spines assessed in the hippocampus and cerebellum are presented in Supplementary Tables 1 and 2.

**Table 1.** Number of Dendritic Spines Characterized and Quantified in the Hippocampus and Cerebellum.

Group (*N* = number of animals)	Hippocampus		Cerebellum—Distal dendrites	
	Apical oblique dendritic spines (*n* = number of cells)	Basal dendritic spines (*n* = number of cells)	Anterior lobe spines (*n* = number of cells)	Central lobe spines (*n* = number of cells)
Reln^+/+^ Veh (*N* = 2)	709 (*n* = 12)	897 (*n* = 12)	425 (*n* = 12)	460 (*n* = 12)
Reln^+/+^ CPO (*N* = 4)	500 (*n* = 14)	623 (*n* = 15)	642 (*n* = 13)	616 (*n* = 12)
Reln^rl/+^ Veh (*N* = 4)	608 (*n* = 11)	486 (*n* = 9)	656 (*n* = 10)	665 (*n* = 11)
Reln^rl/+^ CPO (*N* = 4)	526 (*n* = 8)	536 (*n* = 6)	631 (*n* = 10)	719 (*n* = 11)

### Statistical Analysis

*t*-test comparisons, two-way ANOVA, and Holm-Sidak post hoc analysis were performed in Excel or Sigma Plot.

## Results

Prior studies from our laboratory and others have examined the combinatorial effect of reduced reelin expression and prenatal organophosphate pesticide exposure on behavior and gross brain morphology in mice (Laviola et al., 2006; Mullen et al., 2013). In the current study, we examined the cellular morphology and dendritic spine organization in the adult cortex, hippocampus, and cerebellum, as disruptions in reelin signaling are known to lead to alterations in dendritic spine development and stabilization (Niu et al., 2008; Ventruti et al., 2011)

### Reelin Expression in Adult Animals After Prenatal CPO Exposure

**Figure 1.**
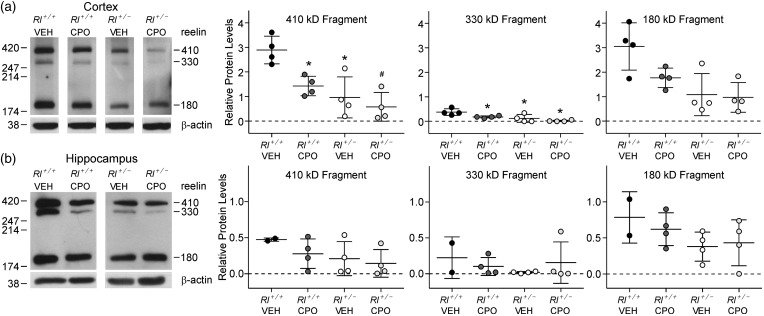
Reelin expression in the adult cerebral cortex and hippocampus. Western blots and reelin fragment quantification from cortical (a) and hippocampal (b) tissue. Three bands representing full-length reelin (410 kDa) and two breakdown products (330 kDa and 180 kDa) were observed; each band was independently quantified compared with ß-actin. Bars in the dot plots are representative of mean ± SD. Significance determined based on comparison to vehicle-treated *Rl^+/+^* animals (*p < .05, ^#^*p* < .01) as calculated by Holm-Sidak post hoc analysis.

Our prior study showed that prenatal exposure to CPO in embryonic *Rl^+/^^−^* mice partially restored reelin expression to levels approaching those seen in *Rl^+/+^* mice (Mullen et al., 2013), possibly due to interference with cleavage of full-length reelin. To determine if this effect persists into the postnatal period, we examined reelin expression in brains at P90. Western blots were used to assess the effects of prenatal CPO exposure on reelin protein levels in adult cerebral and hippocampal cortices (Figure 1). Full-length reelin protein is approximately 410 kDa and upon protein processing results in two smaller fragments at 330 kDa and 180 kDa (Lambert de Rouvroit et al., 1999; Jossin et al., 2007). As expected, overall reelin protein levels were reduced in vehicle-treated adult *Rl^+/^^−^* mice, although surprisingly, this reduction was primarily evident in the cerebral cortex, but not as apparent in the hippocampus. CPO treatment also appeared to reduce the amounts of both full-length and cleaved reelin; these differences were apparent in both the cerebral cortex and the hippocampus but only statistically significant in the cortex. Full-length reelin was reduced in CPO-treated *Rl^+/+^* mice to levels nearly comparable to those seen in vehicle-treated *Rl^+/^^−^* mice, and additional reductions were seen in CPO-treated *Rl^+/^^−^* mice, suggesting that CPO treatment regulates reelin expression independently of the genetic status of the animal. Two-way ANOVA and Holms-Sidak post hoc analysis confirmed the effects of both genotype and CPO treatment in cortical samples.

Comparison of the ratio of the 410 kD fragment to the 180 kD fragment suggested protein increased cleavage in CPO-treated heterozygous animals (410:180 = 0.58) compared with all other groups (WT-Veh 410:180 = 0.94; Het-Veh 410:180 = 0.86; WT-CPO 410:180 = 0.95).

### CPO Treatment Does Not Affect the Presence or Location of GABAergic Interneurons

Reelin is known to be expressed preferentially in GABAergic interneurons in the adult neocortex and hippocampus (Alcantara et al., 1998; Pesold et al., 1998), thus a possible explanation for the observed reduction in reelin protein expression might be a loss of GABAergic interneurons. To determine whether CPO exposure specifically affected reelin-positive GABAergic interneurons, we examined the distribution of these cells in *Balb/C/GAD67^GFP/+^/Rl^Rl^^−^^Orl/+^* adult mice following prenatal CPO exposure. GFP-labeled, GAD67-expressing cells were quantified in both the cerebral cortex (specifically the motor cortex) and the hippocampus. Cortical and hippocampal lamination were unaffected, and the distribution of GAD67-expressing cells was not different with reduced reelin or CPO exposure (see Supplementary Figures 1 and 2). These findings make it unlikely that a reduction or redistribution of GABAergic interneurons is responsible for the observed decreases in reelin protein expression.

### Gross Morphology and Dendritic Spine Distribution in the Hippocampus

**Figure 2.**
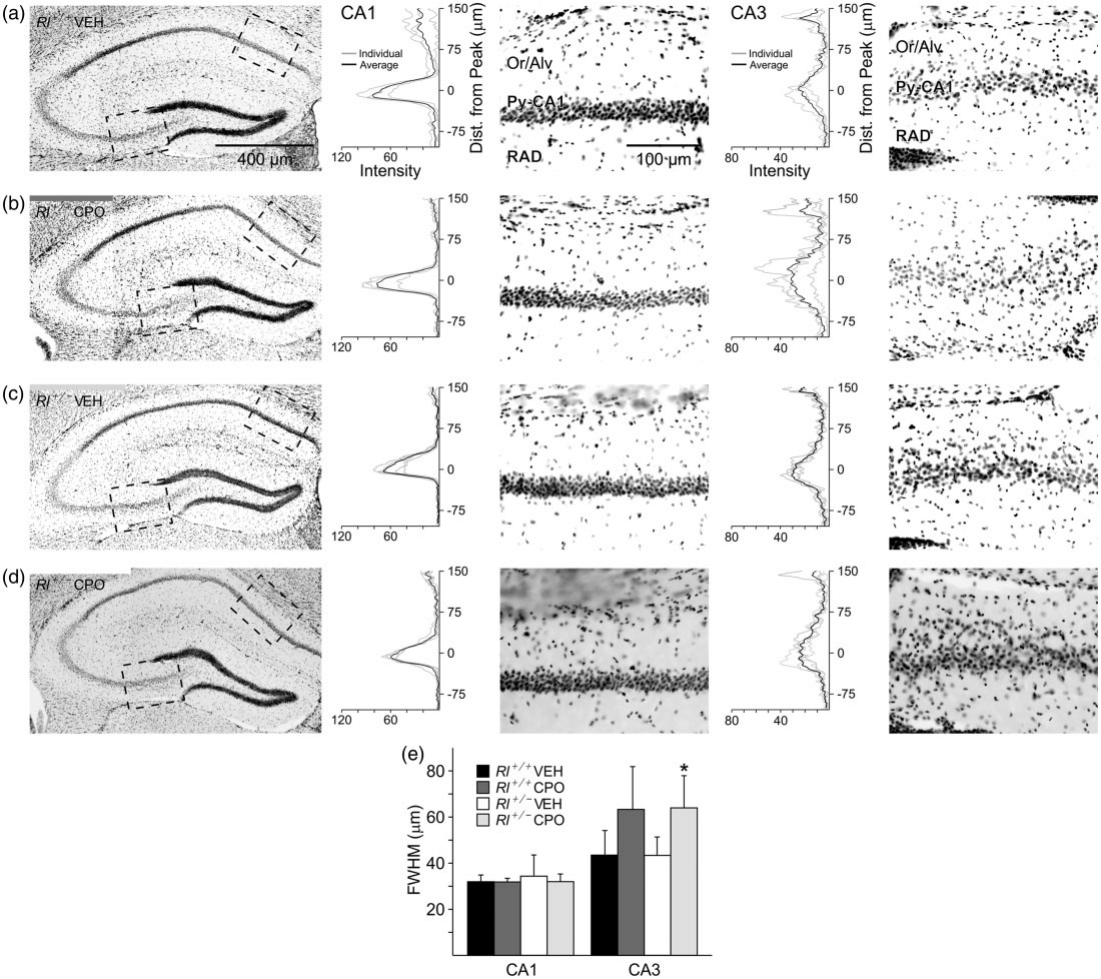
Pyramidal cell distribution in CA1 and CA3 in the hippocampus of vehicle-treated *Rl^+/+^* mice (a), CPO-treated *Rl^+/+^* mice (b), vehicle-treated *Rl^+/^^−^* mice (c), and CPO-treated *Rl^+/^^−^* mice (d) as revealed with DAPI labeling. The black and white pictures are inverse representations of DAPI fluorescence (white = black; black = blue). Boxed regions in CA1 and CA3 were scanned for fluorescence intensity and are represented as histograms adjacent to the high-magnification figures. Histograms illustrate group data, where the black line represents the average and gray lines represent individual animals. (e) Quantification of the half width of the pyramidal cell layer intensity peak for each group. Bars are representative of mean ± SD. Significance determined based on comparison to *Rl^+/+^* Veh (*p < .05) as calculated by Holm-Sidak post hoc analysis.

We examined hippocampal lamination using DAPI-stained tissue, by plotting histograms of the staining intensity in the two pyramidal regions CA1 and CA3 in all four experimental groups (Figure 2). The distribution of DAPI-labeled cells in CA1 was not affected by any experimental conditions, but CA3 appeared to have an expansion of the pyramidal cell layer due to CPO exposure. ANOVA analysis indicated an overall significant difference between treatment groups (*F*_1,10_ = 8.284, *p* = .016), and *t*-test comparisons and post hoc analysis showed that the pyramidal layer in CA3 was significantly widened due to prenatal CPO exposure in *Rl^+/^^−^* animals (*p* < .05); a similar expansion was seen in CPO-treated *Rl^+/+^* animals, though statistical analysis did not indicate significance. As DAPI labeling does not separate neurons and glia, both cell types may be aberrantly positioned.

**Figure 3.**
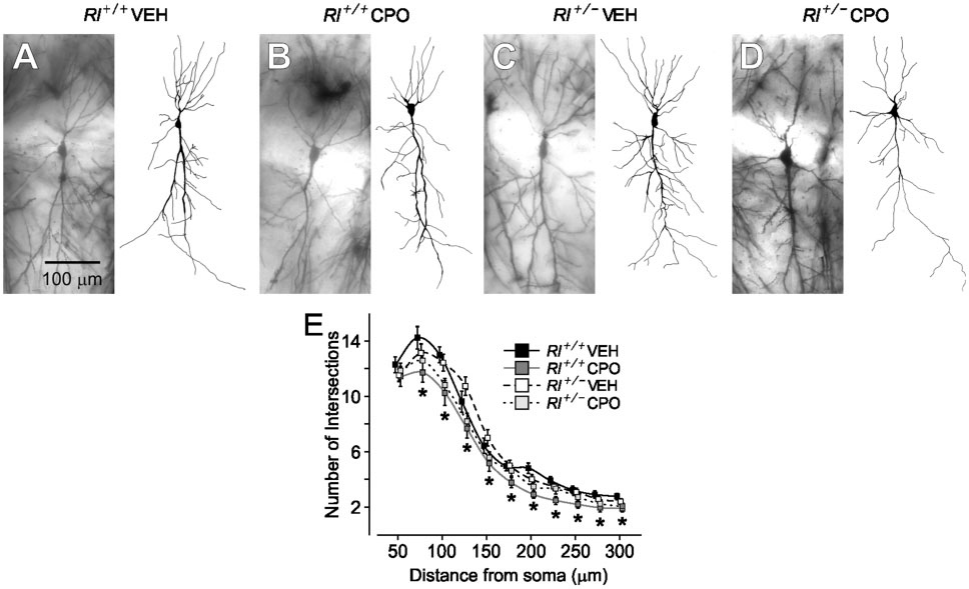
Golgi-stained hippocampal pyramidal cells and representative camera lucida drawings from vehicle-treated *Rl^+/+^* mice (a), CPO-treated *Rl^+/+^* mice (b), vehicle-treated *Rl^+/^^−^* (c), and CPO-treated *Rl^+/^^−^* mice (d). (e) Sholl analysis of camera lucida drawings. Hippocampal pyramidal neurons from CPO-treated *Rl^+/+^* mice have fewer intersections. * indicates significant differences in dendritic complexity as determined by two-way ANOVA. Sholl analysis is represented by mean ± *SE*.

**Figure 4.**
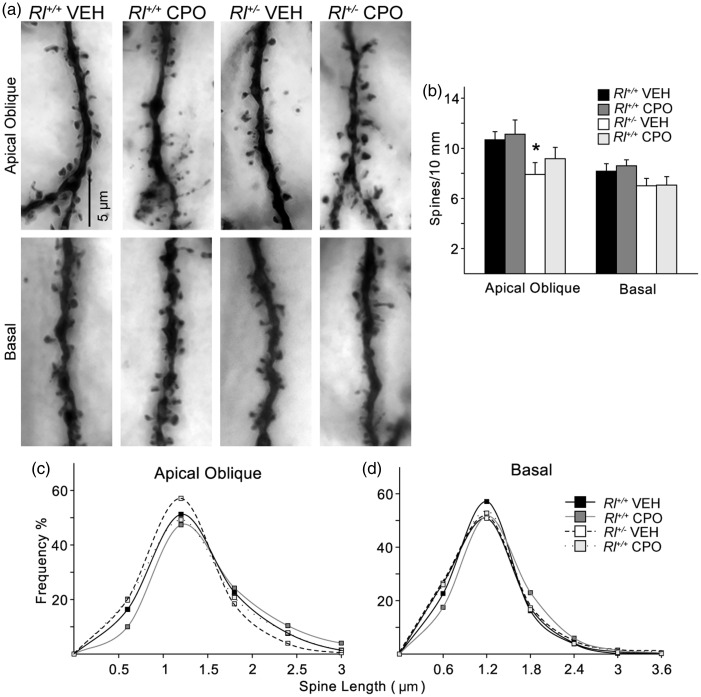
Analysis of dendritic spines on CA1 pyramidal neurons. Apical oblique and basal dendrites of Golgi-impregnated cells (a) were analyzed for total spine density (b) and spine length (c, d). Bars in (b) indicate mean ± *SE*. Significance was determined based on comparison to vehicle-treated Rl^+/+^ cells as calculated by Holm-Sidak post hoc analysis (*p < .05).

**Figure 5.**
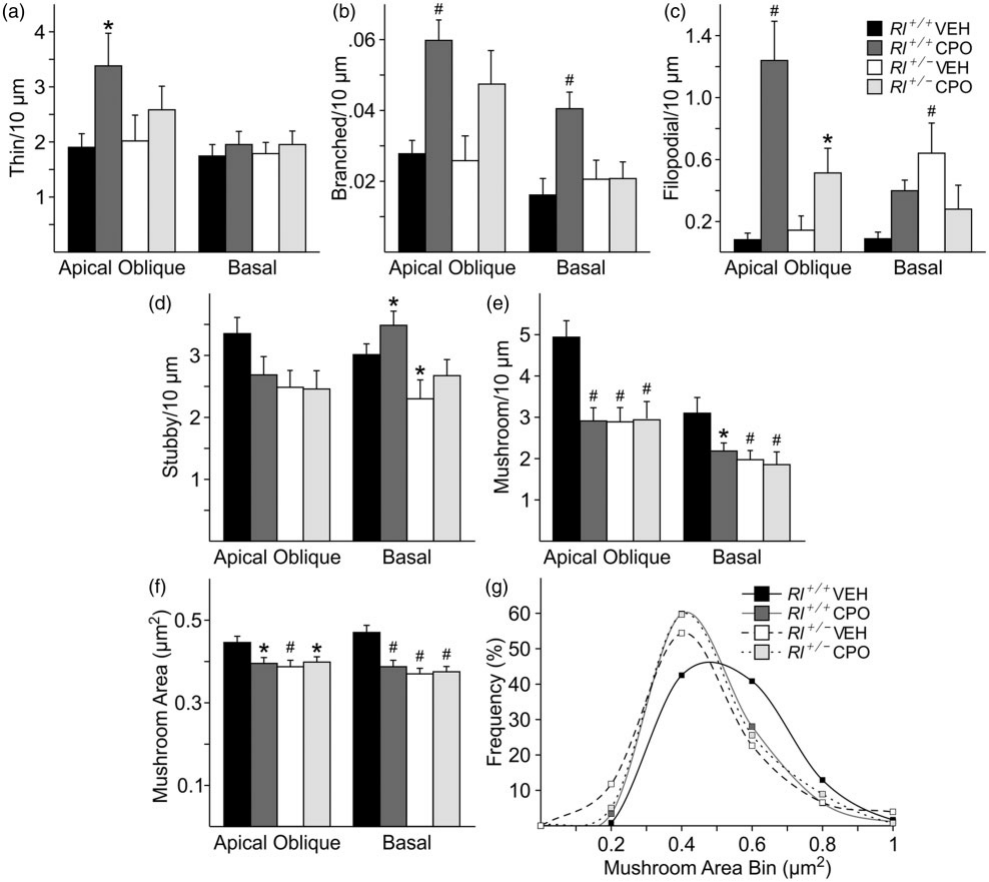
Characterization of hippocampal dendritic spines. Quantification of immature (a, thin; b, branched; c, filopodial) and mature (d, stubby; e, mushroom) spine density and mushroom spine area (f) and frequency (g) on apical oblique and basal hippocampal pyramidal dendrites. Spine density is indicated as number of spines per 10 µm, while mushroom spine area is presented as µm^2^. Significance was determined based on comparison to vehicle-treated *Rl^+/+^* as calculated by Holm-Sidak post hoc analysis (*p < .05; ^#^*p* < .01).

The complexity of dendritic branching of CA1 pyramidal neurons was assessed in camera lucida drawings of Golgi-labeled cells (*Rl^+/+^* Veh, *n* = 21; *Rl^+/+^* CPO, *n* = 21; *Rl^+/^^−^* Veh, *n* = 23; *Rl^+/^^−^* CPO, *n* = 21) and subjected to Sholl analysis (Figure 3). Beginning approximately 75 µm and continuing as far as 300 µm away from the soma, there was a slight but significant decrease in pyramidal cell dendritic complexity due to prenatal CPO exposure in both *Rl^+/+^* and *Rl^+/^^−^* animals. Reelin haploinsufficiency alone had no effect on dendritic branching. We analyzed total spine density and created probability curves of the dendritic spine lengths found on the AO and Bas (Figure 4). Then, we classified the morphology of each of the spines from both the AO and Bas dendrites to further elucidate the effect of each variable (Figure 5). Representative images of Golgi-stained dendritic spines from each experimental condition are shown in Figure 4(a). Quantification and statistics for these analyses are reported in Supplementary Table 1.

**Figure 6.**
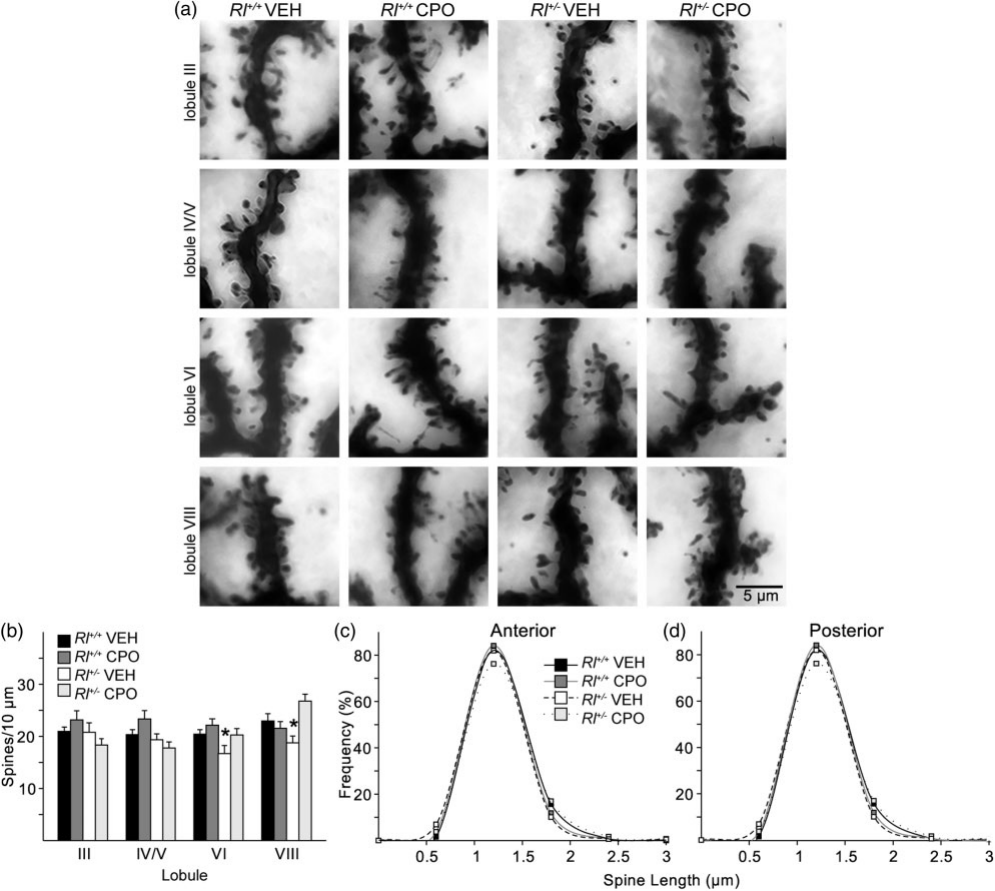
Dendritic spine distribution and length on Purkinje cells in the anterior lobes (Lobule III and IV/V) and posterior lobes (Lobules VI and VIII) of the cerebellum. Spines were visualized on Golgi-impregnated neurons (a) and the quantified as number of spines per 10 µm (b). Spine lengths were also measured and their distribution plotted in the anterior lobules (c) and posterior lobules (d). Bars in B represent mean ± *SE*. Significance determined based on comparison to vehicle-treated *Rl^+/+^* samples and was calculated by Holm-Sidak post hoc analysis (*p < .05).

Total spine density decreased significantly on AO dendrites in vehicle-treated *Rl^+/^^−^* animals (Figure 4(b)), suggesting that reduced reelin expression can affect spine density independently of CPO treatment, similar to data reported in Niu et al. (2008). Average spine length was not significantly affected either by decreased reelin expression or by CPO exposure (Figure 4(c), (d)).

Following the classification established by Lee et al. (2004a), we identified dendritic spines as immature (thin, branched, or filopodial) or mature (mushroom or stubby) spines. We then asked if the distribution of spine types in the hippocampus was affected by either reduced reelin or prenatal CPO treatment. Thin spines were increased on AO dendrites in CPO-treated *Rl^+/+^* animals but were unaffected in all other conditions and on Bas dendrites (Figure 5(a)). Branched spines were increased on AO dendrites in both CPO-treated *Rl^+/+^* and *Rl^+/^^−^* animals and on Bas dendrites in CPO-treated *Rl^+/+^* animals. Filopodial spines were also increased on AO dendrites in both CPO-treated *Rl^+/+^* and *Rl^+/^^−^* animals and on Bas dendrites in vehicle-treated *Rl^+/^^−^* animals and in CPO-treated *Rl^+/+^* and *Rl^+/^^−^* animals. Thus, all three categories of immature spines were affected by loss of reelin expression, CPO exposure, or both (Table 2). Mature stubby spines showed little difference on AO dendrites, although there were slight increases in this spine type on BAS dendrites in CPO-treated *Rl^+/+^* animals and slight decreases in vehicle-treated *Rl^+/^^−^* animals. In contrast, both mushroom spine density and mushroom spine head area were decreased in all treatment groups on both types of dendrites when compared with vehicle-treated *Rl^+/+^* animals. Together, these data suggest an increase in immature spines, and a concomitant decrease in size and density of at least one category of mature spines with reduced reelin or CPO exposure.

### Gross Morphology and Dendritic Spine Distribution in the Cerebellum

Reelin plays a crucial role during the development of the cerebellum, allowing proper migration of Purkinje cells (PC) from midbrain regions out to the rhombic lip (Miyata et al., 1996). It has also been reported that PC loss occurs in both *Rl^+/^^−^* and CPO-treated *Rl^+/+^* animals (Hadj-Sahraoui et al., 1996; Biamonte et al., 2009); thus, we investigated whether a combination of the reduced reelin expression and CPO exposure would affect gross cerebellar organization and PC morphology. We found no significant difference in lobule area and no gross morphological alterations in the PC layers (see Supplementary Figure 3).

**Figure 7.**
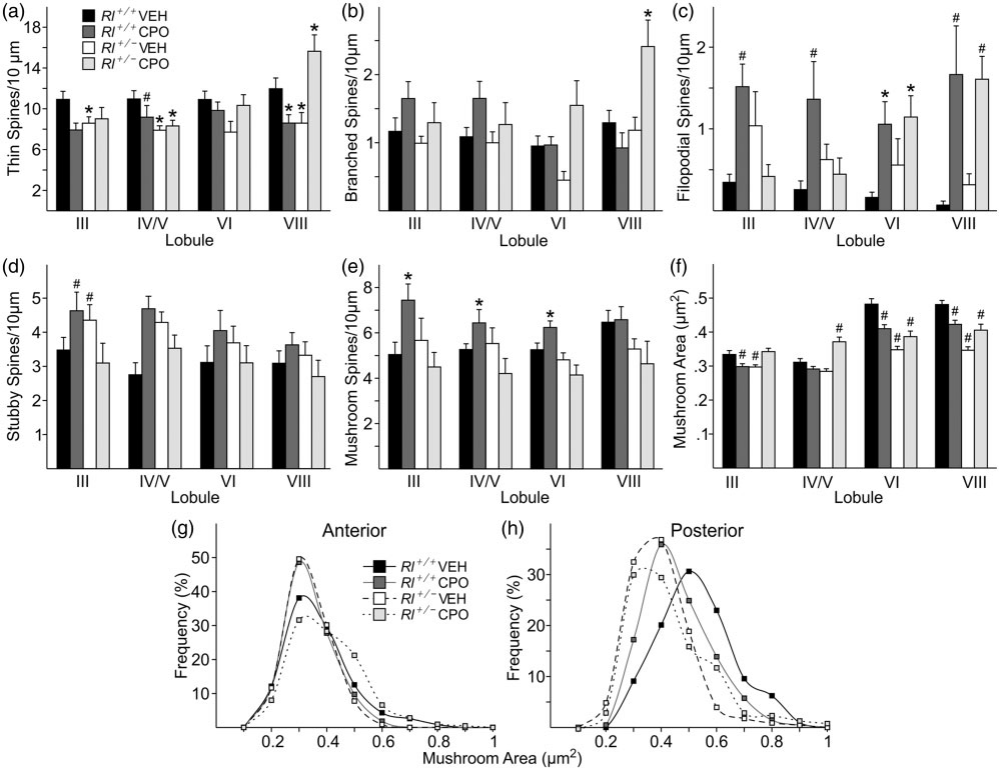
Characterization of cerebellar dendritic spines. Quantification of immature (a, thin; b, branched; c, filopodial) and mature (d, stubby; e, mushroom) spine density and mushroom spine area (f) and frequency (g, h) in four cerebellar regions—Lobule III, Lobule IV/V, Lobule VI, and Lobule VII. Spine density is indicated as number of spines per 10 µm, while mushroom spine area is presented as µm^2^. Bars represent mean ± *SE*. Significance was determined based on comparison to vehicle-treated *Rl^+/+^* as calculated by Holm-Sidak post hoc analysis (*p < .05; ^#^*p* < .01).

We examined PC distal dendrites (Figure 6) in four different lobules of the cerebellum (III, IV/V, VI, and VIII). The anterior lobe, comprising Lobules I–V, is typically associated with information coming from the spinal cord and plays an integral part in sensorimotor systems. The posterior lobe, comprising Lobules VI–IX, receives information primarily from corticopontine pathways associated with higher order cognitive processing (Takahasi et al., 2013). We first characterized the total spine density and created probability curves of the dendritic spine lengths found in the anterior and posterior lobes (Figure 6(c), (d)). We then classified each of the spines from both the anterior and posterior lobe PC dendrites to determine if there were changes in the distribution of the different spine types (Figure 7). All statistics for these analyses are reported in Supplementary Table 2. Representative images of Golgi-stained dendritic spines within each experimental condition and lobule are shown in Figure 6(a).

Total spine density was not different in the anterior lobules in any of the four groups. Spine density was reduced in posterior Lobules VI and VIII in vehicle-treated *Rl^+/^^−^*; however, CPO treatment restored Lobule VI and VIII spine density in *Rl^+/^^−^* to levels similar to vehicle-treated *Rl^+/+^* mice. There were no differences in spine length in any cerebellar region examined.

Spine-type distribution was also examined in the different lobes and lobules (Figure 7 and Table 2). Thin spines were reduced to nearly the same extent in both vehicle-treated *Rl^+/^^−^* and CPO-treated *Rl^+/+^* animals in both the anterior and posterior lobes, and the reduction was statistically significant in all but Lobule VI. Combining both reduced reelin and CPO exposure did not affect thin spine distribution in the anterior lobe, but increased thin spine density in the posterior lobe, primarily in Lobule VIII. Branched spines in the anterior lobe were unaffected either by loss of reelin expression or by CPO exposure. However, in the posterior lobe, CPO treatment increased the number of branched spines in *Rl^+/^^−^* mice compared with *Rl^+/+^* mice. Filopodial spines were the most affected class of spines (Figure 7(c)). Both reduced reelin expression, and CPO treatment increased the number of filopodial spines in all lobules. In the anterior lobe, CPO exposure appeared to have little effect on spines in *Rl^+/^^−^* mice, while in the posterior lobe, CPO-treated *Rl^+/^^−^* mice had a large increase in filopodial spines, significantly above that seen in *Rl^+/+^* animals, and above the level seen in *Rl^+/^^−^* mice as well, suggesting a significant effect of CPO treatment on filopodial spines in the posterior lobe.

Mature spine types appeared to be somewhat less affected than immature spines (Table 2). *Rl^+/^^−^* mice had mild to moderate increases in the density of stubby spines, primarily in the anterior lobe, while stubby spine density was similar to that seen in vehicle-treated *Rl^+/+^* animals in the posterior lobe (Figure 7(d)). CPO treatment alone slightly increased stubby spine concentration, but the combination of reduced reelin expression and CPO exposure returned stubby spine density to normal levels. Mushroom spine density was increased following CPO treatment in *Rl^+/+^* animals in all but Lobule VIII, while decreased reelin expression appeared to have no independent effect on these spines. Combining reduced reelin and CPO exposure either returned mushroom spine density to normal levels or caused a slight overall reduction in mushroom spine density (Figure 7(e)). Mushroom spine cross-sectional area appeared to decrease in *Rl^+/^^−^* and CPO-treated *Rl^+/+^* animals particularly in the posterior lobe (Figure 7(f)), although combining reduced reelin and CPO exposure increased mushroom spine area compared with *Rl^+/^^−^* mice. Interestingly, mushroom spines appeared larger overall in the posterior lobe than in the anterior lobe.

## Discussion

The study reported here demonstrates long-lasting biochemical and anatomical alterations stemming from reduced reelin protein expression or embryonic CPO exposure. We found that embryonic CPO exposure could affect adult levels of reelin protein. In a prior study (Mullen et al., 2013), we demonstrated that both full-length and cleaved reelin protein was decreased in embryonic *Rl^+/^^−^* mice, but that CPO exposure paradoxically restored reelin expression to near wild-type levels. In the current study, we examined whether these changes in reelin protein levels were maintained in adult animals. We found little difference in the absolute level of reelin protein in the four groups, but we did observe an increase in the 180 kD cleavage fragment in CPO-treated heterozygous animals, suggesting increased reelin cleavage. CPO is known to stabilize serine protease activity and this may lead to the increase in reelin cleavage. To our surprise, we found that while embryonic CPO exposure caused in a decrease in reelin protein levels in the cortex of adult *Rl^+/+^* and *Rl^+/^^−^* mice, this was not due to reduced number of reelin-expressing GABAergic interneurons. These results suggest that, at least in selected brain regions, there are substantial long-term biochemical effects resulting from prenatal CPO exposure. Our failure to see similar results in embryonic animals suggests that these biochemical changes unfold slowly, well after the period of CPO exposure is completed. Exposure to high levels of CPO produces acute neurotoxicity, likely stemming from a cholinergic crisis due to inhibition of AChE (Flaskos, 2012), while lower level exposure also results in neurodevelopmental toxicity, possibly through alteration of microtubule structure (Jiang et al., 2010), which may alter cell migration during neurodevelopment.

Our current study also suggests that prenatal CPO exposure affects dendritic spine morphology and distribution, which may contribute to neurodevelopmental disorders. CPO exposure may interact with the reelin signaling pathway in several ways. First, CPO acts as an AChE inhibitor, contributing to acute neurotoxicity at high levels of exposure, and the cholinergic system is known to modulate reelin expression. Nicotine, a cholinergic agonist, can restore reelin mRNA levels in *Rl^+/^^−^* mice in several brain regions (Romano et al., 2013). Second, CPO may act directly on reelin protein itself, by modulating the activity of the enzymes that cleave reelin (Casida and Quistad, 2005), and delayed polyneuropathy has been attributed to exposure to CPO and other organophosphates (Costa, 2006).

Finally, we found significant and lasting effects of reduced reelin expression and CPO exposure on brain morphology. Our prior study found disruptions in gross cellular organization in the olfactory bulb and piriform cortex, the hippocampus, and the cerebellum (Mullen et al., 2013). In the current study, we show region-specific disruptions of hippocampal pyramidal layer organization, along with significant changes in dendritic spine morphology and distribution. We found that CPO exposure, but not reduced reelin expression, affected the placement of pyramidal layer cells in the CA3 region of the hippocampus, although our studies did not distinguish between neurons and glia in this region. Functional studies have suggested that different CA regions subserve varying functions in the hippocampus, with cells in the CA1 regions distinctly responding to spatial information while cells in the CA3 region integrate spatial and nonspatial information (reviewed in Igarashi et al., 2014). These functions may result from the different type of neuronal circuitry at play in each region, with recurrent collateral circuitry evident in CA3, and little cellular interconnectivity in CA1 (Lee et al., 2004b). Our observation of cell displacement in CA3 might then argue that loss of reelin or CPO exposure may affect integration of information in CA3 more strongly than the place recognition modulated by CA1. While we did not directly test spatial memory, which might assist in determining whether hippocampal function is altered following reduced reelin expression and CPO exposure, this would be a worthwhile study for the future.

Hippocampal cellular complexity and hippocampal and cerebellar dendritic spine morphology and distribution were affected both by reduced reelin expression and by CPO exposure, with cellular complexity more strongly affected by reductions in reelin, and dendritic morphology affected in specific regions by CPO exposure. Similar to our previous anatomical studies, combinatorial effects of both reduced reelin and CPO exposure were seen in hippocampal cellular complexity and dendritic spine morphology, with complexity restored and dendritic spine abnormalities partially ameliorated in CPO-exposed *Rl^+/^^−^* animals. Interestingly, there was little evidence of combinatorial interactions on dendritic spine morphology in the cerebellum. Here, CPO exposure alone appeared sufficient to skew the spine profile toward greater representation of immature spines. Our observations suggest an interaction between reduced reelin and CPO exposure in regulating hippocampal cellular morphology, which is more pronounced in AO dendrites, and a singular effect of CPO exposure on cerebellar spines. Prenatal CPO exposure may interfere with spine maturation, possibly through modulation of cholinergic inputs to the hippocampus and cerebellum; manipulation of ACh activity is known to affect synaptic maturation and plasticity (reviewed in Yakel, 2014). However, regardless of their origin, alterations in dendritic spine morphology are widely known to be contributory factors in neurodevelopmental disorders including SZ and ASDs. Our findings point to moderate effects of both loss of reelin and CPO exposure on dendritic spine morphology, although these effects may be quite specific in terms of region or cell type affected. Neurodevelopmental disorders, including SZ and ASDs likely stem from multiple factors; characterization of endophenotypes resulting from each contributing factor may thus provide only a partial characterization of a complex disorder.

## Summary statement

Both the loss of reelin protein expression, caused by genetic mutation, and prenatal pesticide exposure can alter the shape and connectivity of neurons in several brain regions, providing a possible molecular explanation for neurodevelopmental disorders like autism and schizophrenia.

**Table 2.** Summary of Dendritic Spine Analysis.

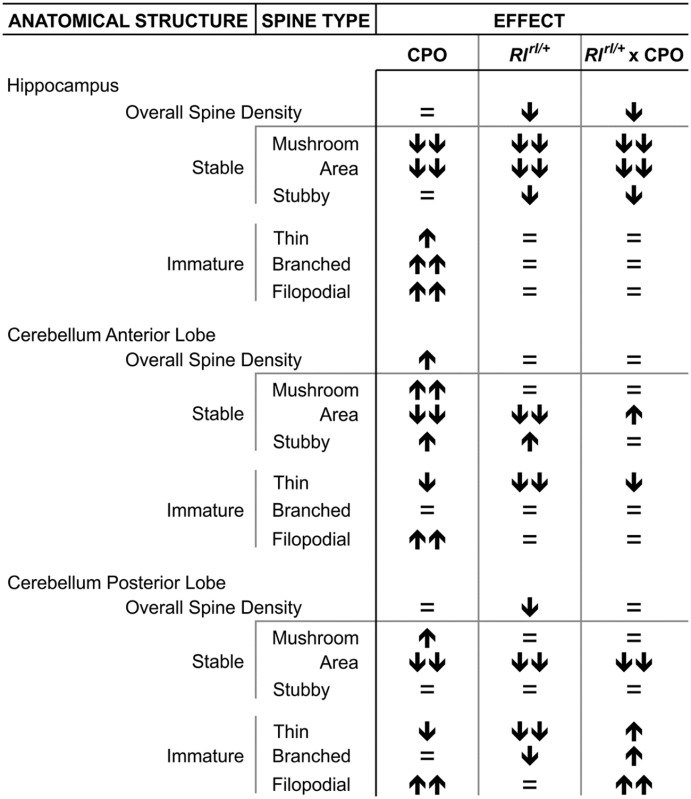	

*Note.* Symbols are changes with respect to Reln^+/+^ Veh animals. No change (=), moderately significant increase (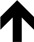) or decrease (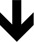), or highly significant increase (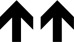) or decrease (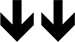).

## Supplementary Material

Supplementary material

## Supplementary Material

Supplementary material
