# Supplementary material for: A Complex Interaction Between Reduced Reelin Expression and Prenatal Organophosphate Exposure Alters Neuronal Cell Morphology
Source: ASN Neuro. 2016 Jun 30;8(3):1759091416656253. doi: 10.1177/1759091416656253 (PMC4962342; doi:10.1177/1759091416656253)
Supplement: Supplementary material [file ASN_656253_Supplementary_Tables.pdf]

Supplementary table 1: Two-way ANOVA data chart for dendritic spine analysis of Hippocampus

	Group	N of mice	n of cells	n of spine[$]	F-statistic, p-value<br>Holm-Sidak Post hoc significant only			
					**Branched Spines**	**Thin Spines**	**Filopodial Spines**	**Total**
**CA1 Apical Oblique**	WT Veh	2	12	--	G: $F_{1,45}$=1.099, p=0.300<br>**T: $F_{1,45}$=15.409, p<0.001**<br>GxT: $F_{1,45}$=0.578, p=0.451<br><br>Post hoc:<br>WT VEH vs WT CPO, p<.0.01[#]<br>HRM VEH vs HRM CPO, p<0.05*	G: $F_{1,45}$=0.567 p=0.455<br>**T: $F_{1,45}$=5.093, p=0.029**<br>GxT: $F_{1,45}$=1.021, p=0.318<br><br>Post hoc:<br>WT VEH vs WT CPO, p<0.05*	**G: $F_{1,45}$=4.547, p=0.038**<br>**T: $F_{1,45}$=7.129, p<0.001**<br>**GxT: $F_{1,45}$=6.382, p=0.015**<br><br>Post hoc<br>WT VEH vs WT CPO, p<0.01[#]<br>WT CPO vs HRM CPO, p<0.01[#]	**G: $F_{1,45}$=6.547, p=0.014**<br>T: $F_{1,45}$=0.849, p=0.362<br>GxT: $F_{1,45}$=0.196, p=0.660<br><br>Post hoc:<br>WT VEH vs HRM VEH, p<0.05*
	WT CPO	3	12	--				
	HRM VEh	3	12	--				
	HRM CPO	3	12	--				
	Total	11	48	--				
					**Mushroom Spine**	**Stubby spine**	**Cross section Area**	
**CA1 Apical Oblique**	WT Veh	2	12	120	**G: $F_{1,45}$=7.127, p=0.011**<br>**T: $F_{1,45}$=6.836, p=0.012**<br>**GxT: $F_{1,45}$=7.498, p=0.009**<br><br>Post hoc:<br>WT VEH vs WT CPO, p<0.01[#]<br>WT VEH vs HRM VEH, p<0.01[#]	G: $F_{1,45}$=3.787, p=0.058<br>T: $F_{1,45}$=1.521, p=0.224<br>GxT: $F_{1,45}$=1.290, p=0.262	G: $F_{1,544}$=3.529, p<0.061<br>T: $F_{1,544}$=1.798, p<0.181<br>**GxT: $F_{1,544}$=4.402, p=0.036**<br><br>Post hoc:<br>WT VEH vs WT CPO, p<0.05*<br>WT VEH vs HRM VEH, p<0.01[#]	
	WT CPO	3	12	133				
	HRM VEh	3	12	162				
	HRM CPO	3	12	133				
	Total	11	48	548				

	Group	N of mice	n of cells	n of spine[$]	F-statistic, p-value<br>Holm-Sidak Post hoc significant only			
					**Branched Spines**	**Thin Spines**	**Filopodial Spines**	Total
**CA1 Basal**	WT Veh	2	12	--	G: $F_{1,44}$=1.833, p=0.183<br>**T: $F_{1,44}$=4.737, p=0.035**<br>**GxT: $F_{1,44}$=4.598, p=0.038**<br><br>Post hoc:<br>WT VEH vs WT CPO, p<.0.01[#]<br>WT CPO vs HRM CPO, p<0.05*	G: $F_{1,44}$=0.0205, p=0.887<br>T: $F_{1,44}$=0.612, p=0.438<br>GxT: $F_{1,44}$=0.0277, p=0.958	G: $F_{1,44}$=2.751, p=0.104<br>T: $F_{1,44}$=0.0385, p=0.845<br>**GxT: $F_{1,44}$=6.602, p=0.014**<br><br>Post hoc:<br>WT VEH vs HET VEH, p<0.01[#]	**G: $F_{1,44}$=5.348, p=0.025**<br>T: $F_{1,44}$=0.174, p=0.679<br>GxT: $F_{1,44}$=0.103, p=0.750
	WT CPO	3	12	--				
	HRM VEh	3	12	--				
	HRM CPO	3	12	--				
	Total	11	48	--				
					**Mushroom Spine**	**Stubby spine**	**Cross section Area**	
**CA1 Basal**	WT Veh	2	12	120	**G: $F_{1,44}$=6.478, p=0.015**<br>T: $F_{1,44}$=3.300, p=0.076<br>GxT: $F_{1,44}$=1.930, p=0.172<br><br>Post hoc:<br>WT VEH vs WT CPO, p<0.05*<br>WT VEH vs HET VEH, p<0.01[#]	**G: $F_{1,44}$=9.587, p=0.003**<br>T: $F_{1,44}$=2.954, p=0.093<br>GxT: $F_{1,44}$=0.0409, p=0.841<br><br>Post hoc:<br>WT VEH vs HET VEH, p<0.05*<br>WT CPO vs HET CPO, p<0.05*	**G: $F_{1,515}$=15.468, p<0.001**<br>**T: $F_{1,515}$=7.278, p=0.007**<br>**GxT: $F_{1,515}$=10.700, p=0.001**<br><br>Post hoc:<br>WT VEH vs WT CPO, p<0.01[#]<br>WT VEH vs HRM VEH, p<0.01[#]	
	WT CPO	3	12	131				
	HRM VEh	3	12	143				
	HRM CPO	3	12	125				
	Total	11	48	519				

[$]: number of spines are for the cross sectional area only G: Main genotype effect; T: Main treatment effect; GXT: Interaction effect
WT- wild type, HRM – Heterozygous reelin mice, VEH- vehicle, CPO-Chlorpyrifos-oxon
Bold: Significant F statistic; * p<0.05; [#] p<0.01

Supplementary table 2: Two-way ANOVA data chart for dendritic spine analysis of cerebellum

	Group	N of mice	n of cells	n of spine$	F-statistic, p-value<br>Holm-Sidak Post hoc significant only			
					**Branched Spines**	**Thin Spines**	**Filopodial Spines**	**Total**
**Lobule III- Immature Spines**	WT Veh	2	10	--	G: $F_{1,34}$=0.978, p=0.33<br>T: $F_{1,34}$=3.047, p=0.090<br>GxT: $F_{1,34}$=0.389, p=0.537	**G: $F_{1,34}$=6.228, p=0.018**<br>T: $F_{1,34}$=0.746, p=0.394<br>GxT: $F_{1,34}$=1.972, p=0.169<br><br>Post hoc:<br>WT VEH vs HET VEH, p<0.05 *	G: $F_{1,34}$=0.923, p=0.344<br>T: $F_{1,34}$=2.615, p=0.115<br>**GxT: $F_{1,34}$=5.005, p=0.032**<br><br>Post hoc:<br>WT VEH vs WT CPO, p<0.01 #<br>WT CPO vs HRM CPO, p<0.05 *	**G: $F_{1,34}$=6.546, p=0.015**<br><br>T: $F_{1,34}$= 0.310, p=0.581<br><br>GxT: $F_{1,34}$=3.184, p=0.083<br><br>Post hoc:<br>WT CPO vs HRM CPO, p<0.01 #
	WT CPO	4	10	--				
	HRM VEh	4	10	--				
	HRM CPO	4	10	--				
	Total	14	40	--				
					**Mushroom Spine**	**Stubby spine**	**Cross section Area**	
**Lobule III- Mature Spines**	WT Veh	2	10	119	G: $F_{1,34}$=2.651, p=0.113<br>T: $F_{1,34}$=3.047, p=0.090<br>**GxT: $F_{1,34}$=0.389, p=0.047**<br><br>Post hoc:<br>WT CPO vs HRM CPO, p<0.05 *	G: $F_{1,34}$=0.282, p=0.559<br>T: $F_{1,34}$=2.712, p=0.109<br>**GxT: $F_{1,34}$=14.223, p<0.001**<br><br>Post hoc:<br>WT VEH vs WT CPO, p<0.01 #<br>WT VEH vs HRM VEH, p<0.01 #<br>WT CPO vs HRM CPO, p<0.01 #	G: $F_{1,451}$=0.143, p=0.705<br>T: $F_{1,451}$=0.255, p=0.614<br>**GxT: $F_{1,451}$=18.925, p<0.001**<br><br>Post hoc;<br>WT VEH vs HRM VEH, p<0.1#<br>WT VEH vs WT CPO, p<0.01#<br>HRM CPO vs HRM VEH,p<0.01#<br>HRM CPO vs WT CPO, p<0.01#	
	WT CPO	4	10	109				
	HRM VEh	4	10	119				
	HRM CPO	4	10	107				
	Total	14	40	454				

	Group	N of mice	n of cells	n of spine$	F-statistic, p-value<br>Holm-Sidak Post hoc significant only			
					**Branched Spines**	**Thin Spines**	**Filopodial Spines**	Total
**Lobule IV/V- Immature Spines**	WT Veh	2	10	--	G: $F_{1,36}$=1.422, p=0.241<br>T: $F_{1,36}$=3.098, p=0.087<br>GxT: $F_{1,36}$=0.166, p=0.686	G: $F_{1,36}$=2.112, p=0.155<br>**T: $F_{1,36}$=6.078, p=0.019**<br>**GxT: $F_{1,36}$=4.257, p=0.046**<br><br>Post hoc<br>WT VEH vs WT CPO, p<0.01#<br>WT VEH vs HRM VEH, p<0.05*	G: $F_{1,36}$=0.594, p=0.446<br>T: $F_{1,36}$=1.087, p=0.304<br>**GxT: $F_{1,36}$=11.374, p=0.002**<br><br>Post hoc:<br>WT VEH vs WT CPO, p<0.1#<br>WT CPO vs HRM CPO, p<0.01#	G: $F_{1,36}$=2.995, p=0.092<br><br>T: $F_{1,36}$=0.00784, p=0.930<br><br>GxT: $F_{1,36}$=2.566, p=0.118
	WT CPO	4	10	--				
	HRM VEh	4	10	--				
	HRM CPO	4	10	--				
	Total	14	40	--				
					**Mushroom Spine**	**Stubby spine**	**Cross section Area**	
**Lobule IV/V- Mature Spines**	WT Veh	2	10	112	G: $F_{1,36}$=2.478, p=0.124<br>T: $F_{1,36}$=0.692, p=0.411<br>**GxT: $F_{1,36}$=5.897, p=0.020**<br><br>Post hoc:<br>WT VEH vs WT CPO, p<0.05*<br>WT CPO vs HRM CPO, p<0.01#	G: $F_{1,36}$=0.435, p=0.514<br>T: $F_{1,36}$=0.0113, p=0.916<br>**GxT: $F_{1,36}$=5.941, p=0.020**<br><br>Post hoc:<br>WT CPO vs HRM CPO, p<0.05 *	**G: $F_{1,433}$=6.938, p=0.009**<br>**T: $F_{1,433}$=10.986, p<0.001**<br>**GxT: $F_{1,433}$=27.848, p<0.001**<br><br>Post hoc;<br>HRM CPO vs HRM VEH,p<0.01#<br>HRM CPO vs WT CPO, p<0.01#	
	WT CPO	4	10	106				
	HRM VEh	4	10	113				
	HRM CPO	4	10	105				
	Total	14	40	436				

$: number of spines are for the cross sectional area only G: Main genotype effect; T: Main treatment effect; GXT: Interaction effect

WT- wild type, HRM – Heterozygous reelin mice, VEH- vehicle, CPO-Chlorpyrifos-oxon

Bold: Significant F statistic; * p<0.05; # p<0.01

Supplementary table 2: Two-way ANOVA data chart for dendritic spine analysis of cerebellum

	Group	N of mice	n of cells	n of spine$^{\$}$	F-statistic, p-value<br>Holm-Sidak Post hoc significant only			
					**Branched Spines**	**Thin Spines**	**Filopodial Spines**	**Total**
**Lobule VI- Immature Spines**	WT Veh	2	10	--	G: $F_{1,35}$=0.840, p=0.840<br>T:$F_{1,35}$=7.446, p=0.010<br>**GxT: $F_{1,35}$=7.050, p=0.012**<br><br>Post hoc:<br>HRM CPO vs HRM VEH, p<0.01$^{\#}$	G: $F_{1,35}$=2.068, p=0.159<br>T: $F_{1,35}$=0.678, p=0.416<br>GxT: $F_{1,35}$=3.883, p=0.057	G: $F_{1,35}$=0.932, p=0.341<br>**T: $F_{1,35}$=8.753, p=0.006**<br>GxT: $F_{1,35}$=0.375, p=0.544<br><br>Post hoc:<br>WT VEH vs WT CPO, p<0.05	**G: $F_{1,35}$=5.077, p=0.0.31**<br><br>**T: $F_{1,35}$=4.632, p=0.038**<br><br>GxT: $F_{1,35}$=0.557, p=0.461<br><br>Post hoc:<br>WT VEH vs HRM VEH, p<0.05*
	WT CPO	4	10	--				
	HRM VEh	4	10	--				
	HRM CPO	4	10	--				
	Total	14	40	--				
					**Mushroom Spine**	**Stubby spine**	**Cross section Area**	
**Lobule VI- Mature Spines**	WT Veh	2	10	108	**G: $F_{1,35}$=14.551, p<0.001**<br>T: $F_{1,35}$=0.215, p=0.646<br>**GxT: $F_{1,35}$=6148, p=0.018**<br><br>Post hoc:<br>WT CPO vs WT VEH, p<0.05*<br>WT CPO vs HRM CPO, p<0.01$^{\#}$	G: $F_{1,35}$=0.128, p=0.722<br>T: $F_{1,35}$=0.110, p=0.742<br>GxT: $F_{1,35}$=2.132, p=0.153	**G: $F_{1,408}$=32.665, p<0.001**<br>T: $F_{1,408}$=1.488, p=0.223<br>GxT: $F_{1,408}$=16.193, p<0.001<br><br>Post hoc:<br>WT VEH vs WT CPO, p<0.01$^{\#}$<br>WT VEH vs HRM VEH, p<0.01$^{\#}$	
	WT CPO	4	10	106				
	HRM VEh	4	10	109				
	HRM CPO	4	10	89				
	Total	14	40	412				

	Group	N of mice	n of cells	n of spine$^{\$}$	F-statistic, p-value<br>Holm-Sidak Post hoc significant only			
					**Branched Spines**	**Thin Spines**	**Filopodial Spines**	Total
**Lobule VIII- Immature Spines**	WT Veh	2	10	--	**G: $F_{1,35}$=7.481, p=0.010**<br>T: $F_{1,35}$=2.937, p=0.095<br>**GxT: $F_{1,35}$=10.114, p=0.003**<br><br>Post hoc:<br>HRM VEH vs HRM CPO, p<0.01$^{\#}$<br>WT CPO vs HRM CPO, p<0.01$^{\#}$	G: $F_{1,35}$=2.658, p=0.112<br>T: $F_{1,35}$=2.658, p=0.112<br>**GxT: $F_{1,35}$=21.398, p<0.001**<br><br>Post hoc:<br>WT VEH vs WT CPO, p<0.05<br>HRM VEH vs HRM CPO, p<0.01$^{\#}$<br>WT VEH vs HRM VEH, p<0.05<br>WT CPO vs HRM CPO, p<0.01$^{\#}$	G: $F_{1,35}$=0772, p=0.783<br>**T: $F_{1,35}$= 18.094, p<0.001**<br>GxT: $F_{1,35}$=0.202, p=0.656<br><br>Post hoc:<br>WT VEH vs WT CPO, p<0.01#<br>HRM VEH vsHRM CPO,p<0.05*	**G: $F_{1,35}$=0.0480, p=0.828**<br><br>T: $F_{1,35}$=2.469, p=0.125<br><br>**GxT: $F_{1,35}$=12.435, p=0.001**<br><br>Post hoc:<br>HRM VEH vsHRM CPO,p<0.01$^{\#}$<br><br>WT VEH vs HRM VEH, p<0.05*<br><br>WT CPO vs HRM CPO, p<0.05*
	WT CPO	4	10	--				
	HRM VEh	4	10	--				
	HRM CPO	4	10	--				
	Total	14	40	--				
					**Mushroom Spine**	**Stubby spine**	**Cross section Area**	
**Lobule VIII- Mature Spines**	WT Veh	2	10	101	**G: $F_{1,35}$=30.968, p=0.005**<br>T: $F_{1,35}$=0.347, p=0.559<br>GxT: $F_{1,35}$=0.890, p=0.352<br><br>Post hoc:<br>WT CPO vs HRM CPO, p<0.01$^{\#}$	G: $F_{1,35}$=0.769 p=0.386<br>T: $F_{1,35}$=0.0120, p=0.913<br>GxT: $F_{1,35}$=2.156, p=0.151	**G: $F_{1,35}$=33.723, p<0.001**<br>T: $F_{1,35}$=000852, p<0.977<br>**GxT: $F_{1,35}$=20.171, p<0.001**<br><br>Post hoc:<br>WT VEH vs WT CPO, p<0.01$^{\#}$<br>HRM VEH vsHRM CPO,p<0.01$^{\#}$<br>WT VEH vs HRM VEH, p<0.01$^{\#}$	
	WT CPO	4	10	103				
	HRM VEh	4	10	119				
	HRM CPO	4	9	103				
	Total	14	39	426				

$^{\$}$: number of spines are for the cross sectional area only G: Main genotype effect; T: Main treatment effect; GXT: Interaction effect
WT- wild type, HRM – Heterozygous reelin mice, VEH- vehicle, CPO-Chlorpyrifos-oxon
Bold: Significant F statistic; * p<0.05; $^{\#}$ p<0.01
