# Supplementary figures and images for: A Complex Interaction Between Reduced Reelin Expression and Prenatal Organophosphate Exposure Alters Neuronal Cell Morphology
Source: ASN Neuro. 2016 Jun 30;8(3):1759091416656253. doi: 10.1177/1759091416656253 (PMC4962342; doi:10.1177/1759091416656253)

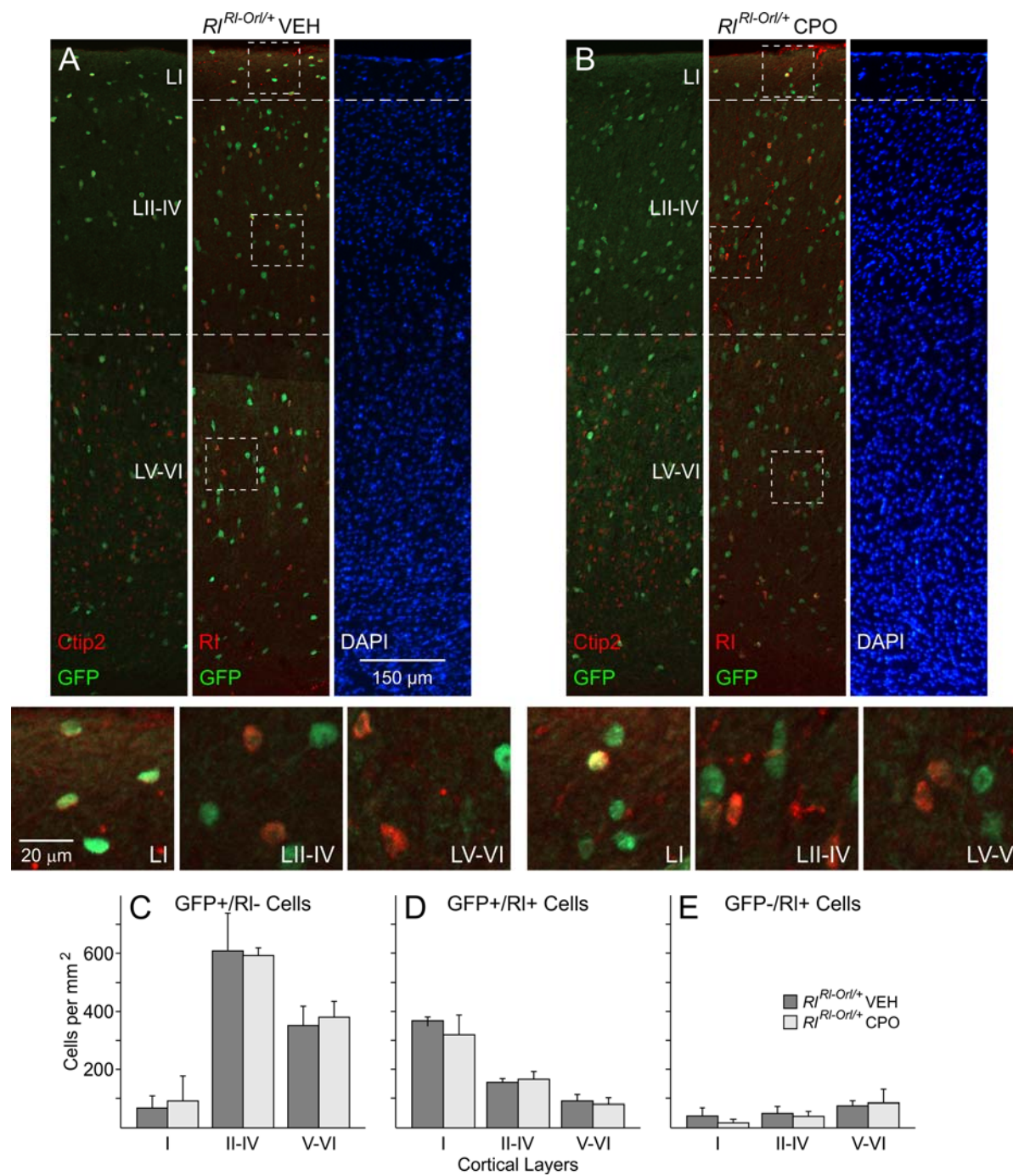

Supplementary Figure 1

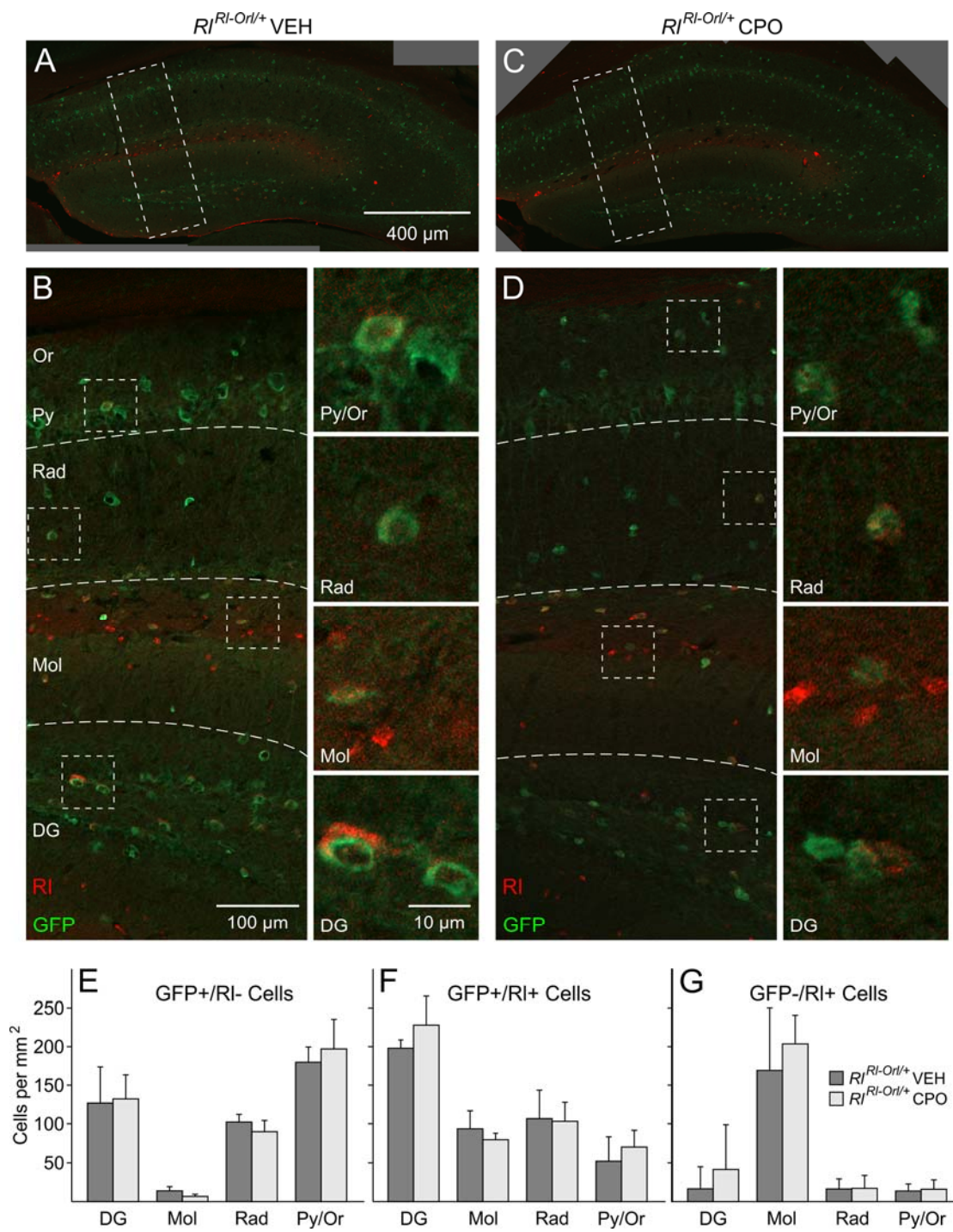

Supplementary Figure 2

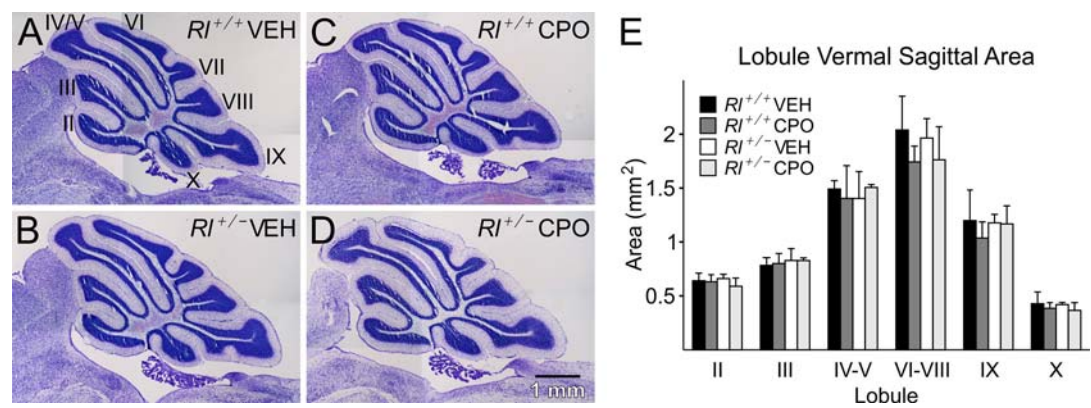

Supplementary Figure 3

Supplement: Supplementary material [file ASN_656253_Supplementary_Figures.pdf]
